# Editorial Comment on “The Impact of Visceral Fat Accumulation on 24‐h Urine Chemistries and Stone‐Recurrence in Patients With Urolithiasis”

**DOI:** 10.1111/iju.70222

**Published:** 2025-09-09

**Authors:** Tomohiro Matsuo, Ryoichi Imamura

**Affiliations:** ^1^ Department of Urology Nagasaki University Graduate School of Biomedical Sciences Nagasaki Japan

This retrospective single‐center study by Deguchi et al. examines how visceral fat area (VFA), quantified on non‐contrast CT, relates to 24‐h urine chemistries and stone recurrence. They report that greater VFA associates with a more lithogenic urinary profile—lower urine pH and higher uric acid, oxalate, phosphate, and sodium—and that VFA was significantly associated with recurrence by log‐rank testing, whereas body mass index and waist circumference were not [1]. These findings align with prior CT‐based work linking higher VFA to lower urine pH, higher urinary sodium, and more uric‐acid stones, although those studies did not assess recurrence [2].

CT‐derived VFA directly captures visceral adiposity and outperforms body mass index or waist circumference as a metabolic proxy, improving construct validity when relating body composition to urine chemistry. Effect sizes appear modest, but in a multifactorial disease, an imaging‐based signal has practical value when paired with standard metabolic evaluation and could inform pragmatic counseling and follow‐up scheduling in busy clinics. As a retrospective analysis, the study is vulnerable to selection bias and residual confounding from diet, hydration, and medications. Generalizability warrants caution: Thresholds such as VFA =100 cm^2^ (used in Japanese metabolic syndrome criteria) may not transfer across demographics. Although VFA tracked with adverse urine chemistries, none of the 24‐h urine parameters differed between patients with and without recurrence, underscoring the multifactorial nature of recurrence [1]. Evidence on urine parameters and recurrence is heterogeneous across cohorts; notably, in young‐adult stone formers, low urinary citrate independently predicts recurrence [3], whereas in our broader cohort, VFA—rather than 24‐h urine indices—was linked to recurrence. This accords with guidelines that individualized metabolic assessment is required [4].

Taken together, VFA is a plausible risk‐stratification adjunct: easy to extract from non‐contrast CT and directionally consistent with the metabolic phenotype seen in 24‐h urine testing [1, 2]. Prospective multicenter studies should calibrate thresholds across populations; test VFA alongside insulin‐resistance indices, inflammatory markers, and dietary exposures; and evaluate whether adding VFA to guideline‐based work‐ups improves recurrence prediction. Standardized, low‐burden analytics for VFA quantification and clear reporting standards (scanner parameters, segmentation, and reproducibility) will be essential for adoption. Deguchi et al. add evidence that visceral adiposity matters for stone biochemistry and recurrence risk; embedding CT‐derived VFA into urolithiasis care, alongside guideline‐driven metabolic evaluation and lifestyle measures, may sharpen identification and intervention for patients at highest risk of another stone.

## Author Contributions

**Tomohiro Matsuo:** writing – original draft, review, and editing. **Ryoichi Imamura:** editing and supervision.

## Conflicts of Interest

The authors declare no conflicts of interest.
